# Symptom or faecal immunochemical test based referral criteria for colorectal cancer detection in symptomatic patients: a diagnostic tests study

**DOI:** 10.1186/s12876-018-0887-7

**Published:** 2018-10-25

**Authors:** Jesús-Miguel Herrero, Pablo Vega, María Salve, Luis Bujanda, Joaquín Cubiella

**Affiliations:** 1 Department of Gastroenterology, Complexo Hospitalario Universitario de Ourense, Ourense, Spain; 2 Instituto de Investigación Biomédica Galicia Sur, Centro de Investigación Biomédica en Red de Enfermedades Hepáticas y Digestivas (CIBERehd),, Ourense, Spain; 3 Donostia Hospital, Biodonostia Institute, University of the Basque Country UPV/EHU, CIBERehd, San Sebastian, Spain

**Keywords:** Colorectal cancer, Faecal immunochemical test, Diagnostic accuracy, Risk stratification

## Abstract

**Background:**

Symptom based referral criteria for colorectal cancer (CRC) detection are the cornerstone of the strategy to improve prognosis in CRC. In 2017, the National Institute for Health and Care Excellence (NICE) updated their referral criteria (2017 NG12). Recently, several studies have evaluated the faecal haemoglobin (f-Hb) concentration in this setting. The aim of this study is to evaluate the diagnostic accuracy of the 2017 NG12 referral criteria and to compare them with the *CG27 referral criteria,* the f-Hb concentration and two f-Hb based prediction model: COLONPREDICT and FAST Score.

**Methods:**

This is a post-hoc diagnostic test study performed within the COLONPREDICT study database (1572 patients, CRC prevalence 13.6%). We assessed symptoms, the 2017 NG12 and CG27 referral criteria and determined the f-Hb before performing a colonoscopy. We compared the discriminatory ability using the area under the curve (AUC) and the sensitivity and specificity at pre-stablished thresholds with the McNemar’s test.

**Results:**

The 2017 NG12 referral criteria discriminatory ability (AUC 0.53; 95% confidence interval- CI 0.49–0.57) was inferior to the CG27 version (AUC 0.59; 95% CI 0.55–0.63; *p* = 0.01), the f-Hb concentration (AUC 0.86; 95% CI 0.84–0-89; *p* < 0.001), the COLONPREDICT Score (AUC 0.92; 95% CI 0.91–0.94; p < 0.001) or the FAST Score (AUC 0.87; 95% CI 0.85–0.89; p < 0.001). The number of patients meeting each criteria were as follows: 2017 NG12 and CG27 = 94.1% and 52.2%; f-Hb ≥20 and ≥ 10 μg/g faeces = 38.6 and 44.3%; COLONPREDICT Score ≥ 5.6 and ≥ 3.2 = 29.4 and 63.2% and FAST Score ≥ 4.50 and ≥ 2.12 = 37.1 and 87.0%. The 2017 NG12 criteria were more sensitive (100%) than the CG27 criteria (68.2%), the f-Hb (≥20 μg/g) (91.2%), the f-Hb (≥10 μg/g) (93.5%), the COLONPREDICT Score (≥5.6) (90.1%) and the FAST Score (≥4.50) (89.8%) (*p* ≤ 0.001) and equivalent to the COLONPREDICT Score (≥3.5) (99.5%) or the FAST Score (≥2.12) (100.0%) (*p* = 1). However, their specificity (6.8%) was significantly lower than any of the evaluated criteria (50.3%, 69.6%, 63.4%, 78.7%, 45.8%, 71.3%, 13.9%; *p* < 0.001).

**Conclusion:**

Referral criteria based on f-Hb measurement, either as a single test or within prediction models, are more accurate than symptom-based referral criteria for CRC detection in symptomatic patients.

## Background

Colorectal cancer (CRC) is the third most common cancer worldwide and the second leading cause of cancer-related death [1]. Two strategies are widely used to detect the disease at an early stage and, thus, improve the prognosis: CRC screening and early diagnosis strategies in symptomatic patients [2, 3]. Although screening programmes have been progressively implemented, most CRC are still detected when symptoms become apparent [4]. In addition, although gastrointestinal symptoms are extremely common in the population, the probability of CRC detection associated with any one symptom is low [5–7]. Thus, risk classification scores have been developed based on symptoms to determine which patients are most at risk of CRC with the aim of reducing this interval between the initial consultation and diagnostic colonoscopy [8, 9].

In this regard, one of the best known referral criteria for CRC detection are the National Institute for Health and Care Excellence (NICE) referral guideline for suspected cancer (CG27) [3]. This referral system has been extensively evaluated showing a low specificity and a variable sensitivity for CRC detection [6, 10–12]. In order to improve these results, the updated version of 2015 (NG12) introduced two significant changes. First, they recommended referral for those symptoms with a positive predictive value of 3% instead of previous 5%. Second, for the first time, testing for occult blood in faeces was recommended in several symptom scenarios with a positive predictive value below 3% [13]. However, the guideline did not recommend any particular method to determine occult blood in faeces.

Faecal immunochemical tests for haemoglobin (FIT) allow for quantitation of faecal haemoglobin concentration (f-Hb). FIT has proven to be the best currently available non-invasive test for CRC screening in asymptomatic individuals and an excellent test for rule-in of CRC and rule-out of significant colonic lesions (SCL) in patients presenting with lower gastrointestinal symptoms [14–21]. On the basis of the available evidence [22], the NICE diagnostic guidance (DG30) recommends the use of FIT with a 10 μg Hb/g faeces to guide referral for colorectal cancer in primary care [23]. However, the effect of the NG12 is not well understood and only one study has evaluated the diagnostic accuracy of this guidance [24]. In July 2017, NG 12 was amended and testing for occult blood in faeces was recommended in patients without rectal bleeding but with unexplained symptoms that do not meet the criteria for a suspected cancer pathway [13].

We have recently developed and validated two f-Hb based prediction models for CRC detection: COLONPREDICT and FAST. The database of the COLONPREDICT Score derivation cohort [25, 26]. is an excellent platform to compare the most widely symptom based referral criteria with the f-Hb concentration based strategies. In this database, an extensive collection of information regarding symptoms as well as several blood and faecal determinations are included. This information allowed us to perform a post hoc analysis in order to evaluate the diagnostic accuracy of the 2017 NG12, compare these criteria with the CG27, the f-Hb concentration and two CRC prediction models based on the f-Hb concentration: COLONPREDICT and FAST Scores [25, 26].

## Methods

### Study design

The current study is a post hoc analysis performed within the COLONPREDICT study: a multicentre, cross-sectional, blinded study of diagnostic tests. The study aimed to create and validate a CRC prediction index based on available biomarkers, clinical and demographical data. We performed this post hoc analysis in the 1572 patients included in the derivation previously described [25].

### Brief description of the COLONPREDICT study

The details of the study have been described extensively elsewhere and are summarized here [25, 26]. We used the Colonoscopy Research into Symptom Prediction questionnaire (CRISP) to record symptoms and demographic data [27]. Based on this questionnaire, they determined if patients met the CG27 referral criteria for CRC detection [3]. f-Hb concentration was assessed using the automated OC-SENSOR MICRO analyser (Eiken Chemical Co., Ltd., Tokyo, Japan). The faeces for the f-Hb determination were collected using the OC-Sensor probe. Moreover, we determined blood haemoglobin (b-Hb) and mean corpuscular volume with a Beckman Coulter Autoanalyzer (Beckman Coulter Inc., CA, USA). Colonoscopy was performed blind for the questionnaire and analytical results.

### 2017 NG12 referral criteria and the f-Hb based prediction models calculation

On the basis of the information obtained from the CRISP questionnaire and the analysis performed (f-Hb, b-Hb and mean corpuscular volume), we determined which of the 2017 NG12 criteria for CRC suspicion were met. Two researchers (JMH and JC) independently decided the equivalence between each NICE criteria and the information collected. Finally, they reached a consensus version. NG12 referral criteria are shown in Table 1 [13]. We considered a positive faecal occult blood test if the f-Hb concentration was ≥10 μg Hb/g faeces.

**Table 1:** Criteria to refer people using a suspected cancer pathway referral for CRC according to the NG12 referral criteria. The number of patients meeting each of the referral criteria is shown

Criteria	Number of patients (*n* = 1572)
Patients ≥40 years with unexplained weight loss and abdominal pain	196 (12.5%)
Patients ≥50 years with unexplained rectal bleeding	811 (51.6%)
Patients ≥60 years with: iron–deficiency anaemia or changes in their bowel habit	890 (56.7%)
Patients with a rectal or abdominal mass	80 (5.1%)
Adults < 50 years with rectal bleeding and any of the following unexplained symptoms or findings: abdominal pain, change in bowel habit, weight loss or iron-deficiency anaemia.	124 (7.9%)
Offer testing for occult blood in faeces to assess for colorectal cancer in adults without rectal bleeding who but with unexplained symptoms that do not meet the criteria for a suspected cancer pathway referral A positive test for occult blood in faeces was considered if the haemoglobin concentration was ≥10 μg Hb/g faeces.	78 (4.9%)
Any of the referral criteria	1479 (94.1%)

COLONPREDICT score is a CRC prediction model based on a multivariable logistic regression analysis [25]. The COLONPREDICT score is based in eleven variables and the mathematical formula is as follows: 0.789 x rectal bleeding + 0.536 x change in bowel habit + 2.694 x rectal mass − 1.283 x benign anorectal lesions + 2.831 x f-Hb ≥20 μg Hb/g faeces + 1.561 x b-Hb (< 10 g/dL) + 0.588 x b-Hb (10–12 g/dL) + 1.511 x CEA ≥3 ng/mL + 0.040 x age (years) + 0.813 x sex (male) -2.073 x previous colonoscopy (last 10 years) -0.849 x continuous treatment with aspirin. It shows a high diagnostic accuracy for CRC detection. Two thresholds have been defined with 90% and 99% sensitivity for CRC: 5.6 and 3.5.

FAST Score is a CRC prediction model based on a multivariable logistic regression analysis [26]. The FAST score is based on three variables and the mathematical formula is as follows: 0 x f-Hb (0) μg Hb/g faeces 0.684 x f-Hb (1, 19) + 2.824 x f-Hb [20, 200) μg Hb/g faeces + 4.184 x f-Hb ≥200 μg Hb/g faeces + 0.031 x age (years) + 0.479 x sex (male). Two thresholds have been defined with 90% and 99% sensitivity for CRC: 4.50 and 2.12.

### Outcomes

The main outcome was CRC detection. According to previous studies evaluating FIT in symptomatic patients, [14–21] we considered significant colonic lesion (SCL) detection as the secondary outcome. We defined SCL as CRC, advanced adenoma (≥10 mm, villous histology, high-grade dysplasia), polyposis (> 10 polyps of any histology, including serrated lesions), histologically confirmed colitis (any aetiology), polyps ≥10 mm, complicated diverticular disease (diverticulitis, bleeding), colonic ulcer and/or bleeding angiodysplasia.

### Statistical analysis

First, we performed a descriptive analysis of the population included in the study. In order to determine differences in diagnostic accuracy between the NG12 referral criteria and the rest of diagnostic criteria, the CG27 referral criteria, the f-Hb concentration, the COLONPREDICT and the FAST score we performed two analysis. First, we determined the number of individuals with a positive result and the sensitivity and the specificity for CRC and SCL detection. We determined if the differences between the sensitivity and the specificity of the NG12 referral criteria and the rest of diagnostic criteria, CG27 referral criteria, the COLONPREDICT and the FAST scores at the pre-stablished thresholds and the f-Hb at a 10 and 20 μg Hb/g faeces concentration threshold, were statistically significant using the McNemar’s test. Finally, we also calculated the positive and negative predictive value (PPV, NPV), the positive and negative likelihood ratios (LR) and the diagnostic Odds Ratio (OR) of all the diagnostic tests. Diagnostic OR is defined as the odds of positivity in subjects with disease relative to the odds in subjects without disease.

In a second step, we evaluated the discriminatory ability using receiver-operating characteristic (ROC) curves for CRC and SCL diagnosis, and we calculated the area under the curve (AUC). We determined whether there were statistically significant differences using the chi-square test of homogeneity of areas. Additionally, we determined if there were differences in the discriminatory ability of each of the diagnostic criteria according to the healthcare level referring the patient to colonoscopy. Primary healthcare referral was determined when a general practitioner was requesting the colonoscopy and secondary healthcare referral was determined when a specialist (gastroenterologist, surgeon..) was requesting the exploration.

We report differences with 95% confidence intervals (CI) and their significance. We consider a *p*-value < 0.05 statistically significant. We carried out the analyses using the IBM SPSS Statistics for Windows version 21.0 (IBM Corp, Armonk, USA) and EPIDAT 3.1 (Dirección Xeral de Saúde Pública, Santiago de Compostela, Spain).

## Results

### Description of the cohort

Among the 1572 patients included in the derivation cohort of the COLONPREDICT, a CRC was detected in 214 (13.6%) patients and a SCL in 463 (29.5%) patients: advanced adenomas in 251 (16.0%), a polyp ≥10 mm with non-adenoma histology in 6 (0.4%), colitis in 36 (2.3%) and other SCLs in 6 (0.4%) patients. Direct referrals from primary care to endoscopic evaluation accounted for 22.9% of the patients included.

As we show in the Table 1, 1,479 out of the 1572 (94.1%) met at least one of the 2017 NG12 referral criteria. In contrast, 52.2% of the patients met any of the CG27 referral criteria, 38.7% had a f-Hb concentration ≥ 20 μg Hb/g faeces, 44.4% had a f-Hb concentration ≥ 10 μg Hb/g faeces, 30.9% had a COLONPREDICT Score ≥ 5.6, 60.5% had a COLONPREDICT Score ≥ 3.5, 37.1% had a FAST Score ≥ 4.50 and 88.0% had a FAST Score ≥ 2.12.

### Analysis of the diagnostic accuracy

The sensitivity of the 2017 NG12 referral criteria for CRC detection reaches 100% at the expense of a low specificity (6.8%). As we show in the Table 2, the sensitivity of the 2017 NG12 referral criteria is superior to the sensitivity of the CG27 referral criteria, the f-Hb (≥20 μg Hb/g and ≥ 10 μg Hb/g faeces), the COLONPREDICT Score at a 5.6 threshold (*p* < 0.001) and the FAST Score at a 4.50 threshold. In contrast, the sensitivity is similar to the COLONPREDICT Score at a 3.2 threshold and the FAST Score at a 2.12 threshold (*p* = 1) and the specificity is inferior to any of the other criteria (*p* < 0.001). The rest of the diagnostic accuracy analysis is displayed in Table 2.

**Table 2:** Evaluation of the diagnostic accuracy of the evaluated strategies for colorectal cancer detection

	Sensititvity^1^	P^2^	Specificity^1^	P^3^	Positive PV^1^	Negative PV^1^	Positive LR^4^	Negative LR^4^	Diagnostic OR^4^
2017 NG12 referral criteria (*n* = 1479)	100% (97.8–100.0)		6.8% (5.6–8.4)		14.5% (12.8–16.5)	100% (95.0–100.0)	1.07 (1.06–1.09)	NE	NE
CG27 referral criteria (*n* = 821)	68.2% (61.5–74.3)	*p* < 0.001	50.3% (47.6–53.0)	p < 0.001	17.8% (15.3–20.6)	91.0% (89.0–93.0)	1.4 (1.2–1.5)	0.6 (0.5–0.8)	2.2 (1.6–2.9)
f-Hb ≥ 20 μg Hb/g faeces (*n* = 607)	91.2% (86.3–94.4)	*p* < 0.001	69.6% (67.1–72.0)	p < 0.001	32.3% (28.6–36.2)	98.0% (96.9–98.8)	3.0 (2.7–3.3)	0.1 (0.08–0.2)	23.6 (14.5–38.3)
f-Hb ≥ 10 μg Hb/g faeces (*n* = 696)	93.5% (89.1–96.3)	*p* = 0.001	63.4% (60.7–66.0)	p < 0.001	28.9% (25.6–32.4)	98.4 (97.2–99.1)	2.6 (2.4–2.8)	0.1 (0.06–0.2)	24.8 (14.3–43.2)
COLONPREDICT Score ≥ 5.6 (*n* = 463)	90.1% (85.1–93.6)	p < 0.001	78.7% (76.4–80.9)	p < 0.001	40.7% (36.2–45.3)	98.0% (96.9–98.7)	4.2 (3.8–4.7)	0.1 (0.08–0.2)	33.8 (21.1–54.0)
COLONPREDICT Score ≥ 3.2 (*n* = 994)	99.5% (97.0–100.0)	1	45.8% (43.1–48.2)	*p* < 0.001	22.9% (20.3–25.8)	99.8% (98.9–100.0)	1.8 (1.7–1.9)	0.01 (0.0–0.07)	179 (25–1280)
FAST Score ≥ 4.50 (*n* = 583)	89.8% (84.7–93.3)	*p* < 0.001	71.3% (68.8–73.7)	*p* < 0.001	33.2% (29.4–37.2)	97.8% (96.6–98.6)	3.13 (2.84–3.44)	0.14 (0.10–0.21)	21.8 (13.8–34.4)
FAST Score ≥ 2.12 (*n* = 1383)	100.0% (97.8–100.0)	*p* = 1	13.9% (12.1–15.9)	*p* < 0.001	15.6% (13.7–17.6)	100% (97.5–100.0)	1.16 (1.14–1.19)	NE	NE

^1^Values are expressed as percentages and its 95% confidence interval

^2^Significance of the sensitivity differences when compared with the NG12 referral criteria in McNemar’s test. Differences with *p* < 0.05 are considered statistically significant

^3^Significance of the specificity differences when compared with the NG12 referral criteria in McNemar’s test. Differences with *p* < 0.05 are considered statistically significant

^4^Values are expressed as absolute numbers and its 95% confidence interval

*PV*, predictive value; *LR*, likelihood ratio; *OR*, odds ratio; *f-Hb*, faecal haemoglobin; *NE*, non evaluable

On the other hand, 2017 NG12 referral criteria allows the diagnosis of 98.9% of SCL. As in the diagnostic accuracy for CRC detection, the specificity is extremely low (7.9%). As we show in the Table 3, the sensitivity of the 2017 NG12 referral criteria is similar to the FAST Score at a 2.12 threshold (p = 1) and superior the rest of the evaluated criteria. In contrast, the specificity of the 2017 NG12 criteria is inferior to any of the additional criteria evaluated (p < 0.001). We show the PPV, NPV, positive and negative LR and the diagnostic OR in Table 2.

**Table 3:** Evaluation of the diagnostic accuracy of the evaluated strategies for significant colonic lesion detection

	Sensititvity^1^	P^2^	Specificity^1^	P^3^	Positive PV^1^	Negative PV^1^	Positive LR^4^	Negative LR^4^	Diagnostic OR^4^
2017 NG12 referral criteria (*n* = 1479)	98.9% (97.3–99.6)		7.9% (6.4–9.7)		30.9% (28.6–33.4)	94.6% (87.3–98.0)	1.07 (1.05–1.10)	0.14 (0.06–0.3)	7.9 (3.2–19.5)
CG27 referral criteria (*n* = 821)	58.3% (53.6–62.8)	p < 0.001	50.3% (47.3–53.3)	p < 0.001	32.9% (29.7–36.2)	74.3% (71.0–77.4)	1.17 (1.07–1.29)	0.83 (0.73–0.94)	1.4 (1.1–1.7)
f-Hb ≥ 20 μg Hb/g faeces (*n* = 607)	74.2% (70.0–78.1)	p < 0.001	76.1% (73.5–78.6)	p < 0.001	56.5% (52.4–60.5)	87.6% (85.3–89.6)	3.11 (2.76–3.50)	0.34 (0.29–0.40)	9.2 (7.1–11.8)
f-Hb ≥ 10 μg Hb/g faeces (*n* = 696)	79.4% (75.4–83.0)	p < 0.001	70.2% (67.4–72.9)	p < 0.001	52.7% (49.0–56.5)	89.1% (86.8–91.0)	2.67 (2.41–2.95)	0.29 (0.24–0.35)	9.1 (7.0–11.8)
COLONPREDICT Score ≥ 5.6 (*n* = 463)	64.2% (59.5–68.5)	p < 0.001	83.1% (80.7–85.2)	p < 0.001	61.4% (56.9–65.8)	84.7% (82.3–86.7)	3.79 (3.27–4.40)	0.43 (0.38–0.49)	8.8 (6.8–11.3)
COLONPREDICT Score ≥ 3.2 (*n* = 994)	88.7% (85.3–91.4)	p < 0.001	51.3% (48.3–54.3)	p < 0.001	43.3% (40.1–46.6)	91.5% (89.0–93.6)	1.82 (1.70–1.95)	0.22 (0.17–0.29)	8.3 (6.0–11.3)
FAST Score ≥ 4.50 (*n* = 583)	72.7% (68.4–76.7)	p < 0.001	77.8% (75.2–80.2)	p < 0.001	57.8% (53.7–61.9)	87.2% (84.9–89.2)	3.28 (2.90–3.71)	0.35 (0.30–0.41)	9.4 (7.3–12.0)
FAST Score ≥ 2.12 (*n* = 1383)	97.8% (95.9–98.9)	p = 1	16.1% (14.0–18.4)	p < 0.001	32.8% (30.3–35.3)	94.7% (90.2–97.3)	1.17 (1.13–1.2)	0.13 (0.07–0.25)	8.7 (4.5–16.5)

^1^Values are expressed as percentages and its 95% confidence interval

^2^Significance of the sensitivity differences when compared with the NG12 referral criteria in McNemar’s test. Differences with *p* < 0.05 are considered statistically significant

^3^Significance of the specificity differences when compared with the NG12 referral criteria in McNemar’s test. Differences with *p* < 0.05 are considered statistically significant

^4^Values are expressed as absolute numbers and its 95% confidence interval

*PV*, predictive value; *LR*, likelihood ratio; *OR*, odds ratio; *f-Hb*, faecal haemoglobin; *NE*, non evaluable

### Analysis of the discriminatory ability

**Fig. 1:**
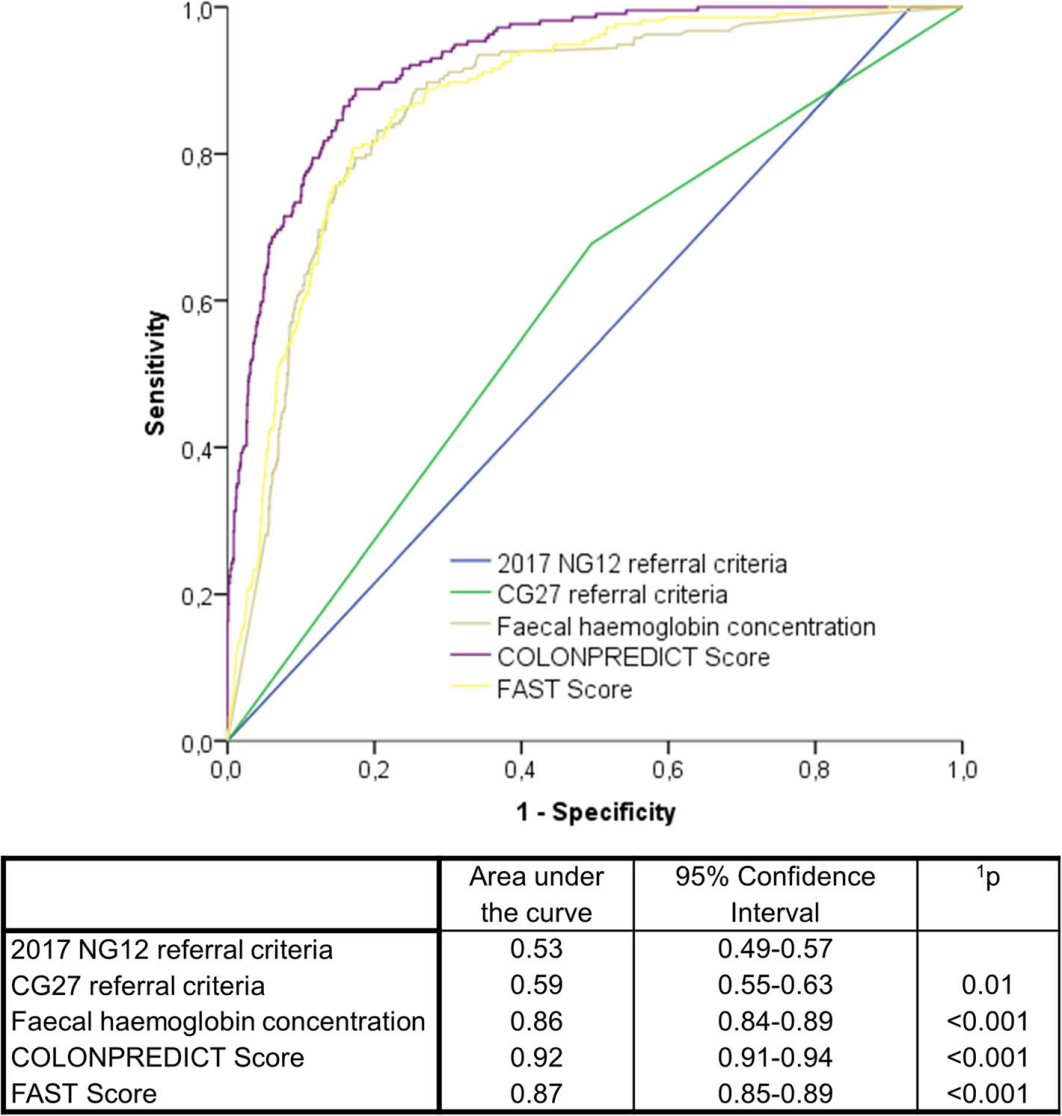
ROC curves of the NICE referral criteria, faecal haemoglobin concentration and the COLONPREDICT and FAST Scores for colorectal cancer detection. The area under the curve of the ROC curves are shown. ^1^Significance of the discriminatory ability differences when compared with the NG12 referral criteria in Chi square homogeneity test. Differences with *p* < 0.05 are considered statistically significant. ROC, Receiver-operating characteristics; NICE, National Institute for Health and Care Excellence

**Fig. 2:**
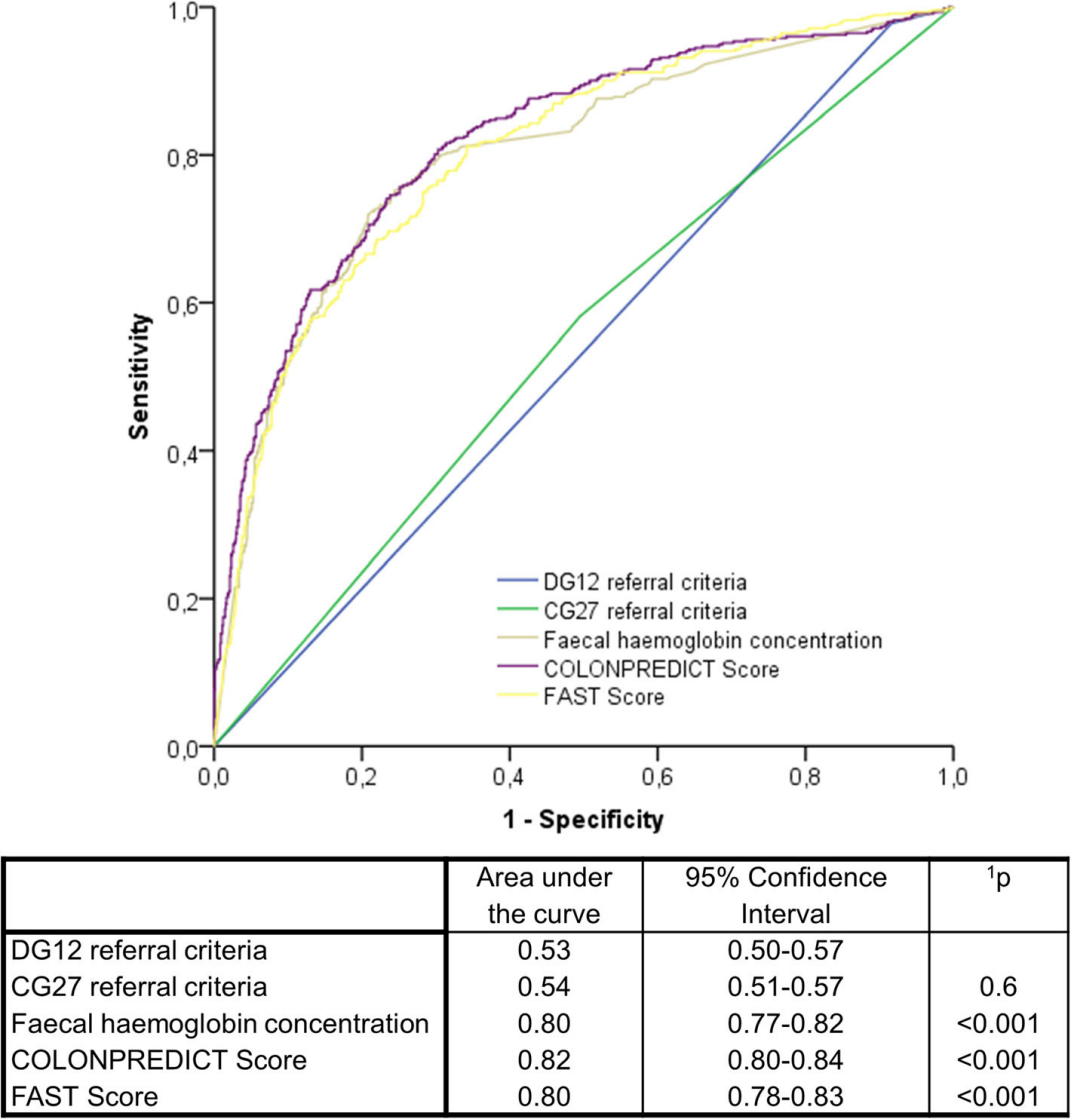
ROC curves of the NICE referral criteria, faecal haemoglobin concentration and the COLONPREDICT and FAST Scores for significant colonic lesion detection. The area under the curve of the ROC curves are shown. ^1^Significance of the discriminatory ability differences when compared with the NG12 referral criteria in Chi square homogeneity test. Differences with p < 0.05 are considered statistically significant. ROC, Receiver-operating characteristics; NICE, National Institute for Health and Care Excellence

The analysis of the discriminatory ability for CRC detection of the NICE referral criteria, the f-Hb concentration, the COLONPREDICT and the FAST Score is shown in Fig. 1. The discriminatory ability of the 2017 NG12 referral criteria is inferior to any of the evaluated criteria in the Chi-square homogeneity test comparison of AUC. Additionally, we found no differences in the performance of each diagnostic test in the evaluation of the discriminatory ability according to the healthcare referring the patient to colonoscopy: 2017 NG12 referral criteria (primary = 0.53, 95% CI 0.46–0.60; secondary = 0.53, 95% CI 0.48–0.58; *p* = 0.1), CG27 referral criteria (primary = 0.60, 95% CI 0.54–0.66; secondary = 0.59, 95% CI 0.55–0.63; *p* = 0.7), f-Hb concentration (primary = 0.85, 95% CI 0.80–0.89; secondary = 0.86, 95% CI 0.83–0.89; *p* = 0.5), COLONPREDICT Score (primary = 0.90, 95% CI 0.86–0.94; secondary = 0.93, 95% CI 0.91–0.95; p = 0.1), FAST Score (primary = 0.84, 95% CI 0.79–0.89; secondary = 0.88, 95% CI 0.85–0.90; p = 0.1). Fig. 2 shows the discriminatory ability for SCL detection of the diagnostic tests evaluated. The discriminatory ability of the 2017 NG12 referral criteria is similar to the CG27 referral criteria and inferior to the rest of the evaluated criteria in the Chi-square homogeneity test comparison of AUC.

## Discussion

### Summary

We have evaluated the diagnostic accuracy of the 2017 NG12 referral criteria for suspected CRC. As we clearly show, these updated criteria are more sensitive than CG27 version. However, they produce a marked increase in the number of patients meeting them and a reduction in the specificity. Furthermore, we had the opportunity to compare them with the f-Hb concentration and two f-Hb based prediction model. As we clearly show, both diagnostic tools have a higher discriminatory ability than the NICE referral criteria.

### Strengths and limitations

We have used a wide cohort of consecutive patients referred to colonoscopy due to gastrointestinal symptoms. Patients were evaluated homogenously using a symptom questionnaire and several analytics, including a FIT, were performed before colonoscopy. This questionnaire allowed us to gather all the details regarding type of symptoms, duration and evolution. Thus, we could evaluate the 2017 NG12 referral criteria for suspected CRC for the first time. Furthermore, we have been able to evaluate the use of FIT, with the 10 μg Hb/g faeces, as recommended in the DG30 [23]. On the other hand, our study has several limitations that must be taken in consideration. We cannot exclude a risk of bias of selection, as long as the symptomatic patients included in the study were previously selected for colonoscopy evaluation. However, we have included all consecutive patients referred both from primary and secondary healthcare to colonoscopy.

### Comparison with existing literature

The update of the NICE referral criteria is based on reducing the PPV threshold required to refer patients from primary care using a suspected cancer pathway referral. The guideline development group agreed to use a threshold value of 3% PPV to underpin their recommendations [13]. So, those patients with symptoms (i.e ≥ 40 years with unexplained weight loss) with a PPV > 3% for CRC should be referred for further testing. Our results clearly demonstrate that this strategy increases the sensitivity for CRC detection in comparison with previous criteria. However, these criteria certainly introduce a risk of over investigation. In fact, what our analysis confirms is that the discriminatory ability of any group of symptoms for CRC detection is suboptimal [6].

An additional innovation of the 2017 NG12 referral criteria is the inclusion of the faecal occult blood test in the evaluation of symptomatic patients. However, its use is only limited to patients without rectal bleeding and with unexplained symptoms that do not meet the criteria for a suspected cancer pathway referral [13]. Our results confirm the data previously published: the f-Hb concentration measured with a FIT shows a higher discriminatory ability for CRC detection than the NICE referral criteria [14–19]. So, probably, the strategy for the evaluation of the risk of CRC detection in symptomatic patients should be based on the f-Hb concentration irrespective of symptoms. Actually, in the COLONPREDICT Score, patients with a f-Hb concentration ≥ 20 μg Hb/g faeces have 17.0 times more risk of CRC detection. In contrast, patients with rectal bleeding or a change in bowel habit have 2.2 and 1.7 times more risk of CRC detection, respectively [25].

Recently, an article has evaluated the diagnostic accuracy of the 2017 NG12 referral criteria for CRC and SCL detection and compared these criteria with the f-Hb concentration [24]. This study used the database of three diagnostic tests studies evaluating FIT in symptomatic patients [15, 17, 19]. and shows that the discriminatory ability of the 2017 NG12 referral criteria are inferior to the f-Hb concentration. This cohort has significant differences with ours: the prevalence of symptoms related to CRC diagnosis, rectal bleeding, changes in bowel habit, iron-deficiency anaemia or rectal mass, is inferior as well as the prevalence of CRC or SCL. Probably, these differences are responsible for the differences in the number of patients that meet 2017 NG12 referral criteria and in the inferior discriminatory ability documented in our study. However, are results are consistent in the comparison of the 2017 NG12 referral criteria with the f-Hb concentration.

### Implications for research and/or practice

One of the main lessons learned in these years from the CRC screening programs is that lack of symptoms or the presence of non-specific symptoms do not exclude a CRC in adult population. Up to 20% of the incident CRC are detected in asymptomatic patients within a CRC screening program based in a guaiac faecal occult blood test [4]. So, the strategies for CRC detection in symptomatic patients should determine which patients require urgent referral, which require a normal referral and, finally, in what situations no additional evaluation is required. The NICE referral criteria only determine the scenarios where an urgent referral is required. Due to the increased discriminatory ability of the FIT for CRC, either the f-Hb concentration alone or a f-Hb based prediction model can allow to establish these three risk groups with different diagnostic strategies. As we have recently proposed, at least 90% of CRC should be detected in a high-risk group, requiring a fast-track referral to colonoscopy. In contrast, in a low-risk group, where no additional explorations are required, the probability of a missing CRC should be well below 1%, so that the risk of CRC is balanced with the risk of colonoscopy complications, mainly perforation [28].

## Conclusions

To conclude, the discriminatory ability of any symptom based criteria is limited when compared with a f-Hb concentration based strategy. An urgent evaluation of the diagnostic accuracy of FIT in symptomatic patients attending primary care is required.

## Abbreviations

AUC: Area under the curve
CEA: Carcinoembryonic antigen
CI: Confidence interval
CRC: Colorectal cancer
CRISP: Colonoscopy Research into Symptom Prediction questionnaire
f-Hb: faecal haemoglobin
FIT: Faecal immunochemical test
LR: Likelihood ratio
NICE: National Institute for Health and Care Excellence
NPV: Negative predictive value
OR: Odds Ratio
PPV: Positive predictive value
ROC: Receiver-operating characteristic
SCL: Significant colonic lesion
